# Enhancing Nursing Students’ Attitudes Toward Older Adults Through an Artificial Intelligence Virtual Simulation: A Mixed-Method Design

**DOI:** 10.3390/nursrep14040279

**Published:** 2024-12-03

**Authors:** Anne White, Mary Beth Maguire, Austin Brown

**Affiliations:** 1 Wellstar School of Nursing, Kennesaw State University, Kennesaw, GA 30144, USA; mbm2332@gmail.com; 2 School of Data Science and Analytics, Kennesaw State University, Kennesaw, GA 30144, USA; abrow708@kennesaw.edu

**Keywords:** artificial intelligence, simulation training, nursing education, geriatrics, attitudes survey

## Abstract

Background/Objectives: The projected increase from 58 million older adults in 2022 to 82 million by 2050 in the United States highlights the urgency of preparing nursing students to care for this aging population. However, studies reveal negative attitudes among nursing students toward older adults. A three-phased educational intervention that included an artificial intelligence (AI)-driven virtual simulation was implemented to address this. AI-generated simulations promise to expose marginalized groups and strengthen future nurses’ knowledge, skills, and attitudes. Methods: A convergent mixed-method design was used to measure the change in nursing students’ attitudes toward older adults, as measured by the UCLA Geriatrics Attitudes Survey and a Guided Reflection survey after participating in an Artificial Intelligence in Education learning event (*n* = 151). Results: The results indicate that post-intervention scores (M = 35.07, SD = 5.34) increased from pre-intervention scores (M = 34.50, SD = 4.86). This difference was statistically significant at the 0.10 significance level (t = 1.88, *p* = 0.06). The qualitative analysis indicated that the attitudes impacted were challenging and overcoming ageism, increased empathy and patience, and enhanced communication skills. Conclusions: Utilizing artificial intelligence technology during educational events effectively yields measurable learning outcomes. Cultivating positive attitudes toward older adults is essential for competent care in an aging society. This study was prospectively approved by the university’s Institutional Review Board (IRB) on 30 July 2021, IRB-FY22-3.

## 1. Introduction

The projected increase from 58 million older adults in 2022 to 82 million by 2050 in the United States (US) highlights the urgency of preparing nursing students to care for this aging population [1]. The National Hartford Center of Gerontological Nursing Excellence [2] has published guidelines on the competencies required for nursing care of older adults, emphasizing the importance of integrating these into the nursing curricula. However, there is a significant gap in education regarding geriatric care within many nursing programs, which has profound implications for the quality of care older adults receive [3]. Studies have shown that nursing students often enter the field with minimal exposure to geriatric care, leading to a lack of confidence and competence when working with older adults [4,5]. As the population of older adults continues to grow, it becomes imperative to address these educational gaps to ensure that future nurses are well-equipped to provide compassionate and effective care.

Ageism and negative attitudes toward older adults are prevalent in undergraduate nursing students and exacerbate senior care challenges [6,7]. These biases can significantly affect the outcomes of older patients, leading to disparities in treatment and care [8,9,10]. Therefore, addressing these attitudes early in nursing education is crucial to fostering a more respectful and empathetic approach to older adult care. Integrating comprehensive geriatric education into nursing curricula can significantly enhance students’ knowledge and skills. Dahlke et al. [11] found that educational interventions included in theory or clinical courses or a combination of both changed nursing students’ negative attitudes toward older adults. However, few significantly impacted nursing students’ willingness to work with older people. In a systematic review and meta-analysis, Burns et al. [12] found that gerontological educational interventions combined with intergenerational contact significantly reduced ageism outcomes related to attitudes, knowledge, and comfort toward older adults. Other researchers found that incorporating service learning and simulation into the nursing curricula significantly influenced baccalaureate nursing students’ attitudes toward older adults [13].

Cheng et al. [3] found that simulation-based learning significantly improved nursing students’ attitudes toward older adults. Also, the researchers found that simulation plus traditional methods are most effective when considering knowledge and attitude [3]. Finally, a study by Venables et al. [14] explored factors influencing nursing students’ attitudes toward older adults and the tools used to measure these attitudes. The researchers found that positive attitudes were linked to advanced years of study, professional values, education on elder care, knowledge about aging, and personal interactions with older adults. The review also highlighted the necessity for a modern, nursing-specific instrument to assess these attitudes. However, most researchers emphasized the need to continue researching interventions to decrease ageism [3,12,13,14].

The literature on the use of AI in nursing education highlights several key findings and recommendations. De Gagne [15] discusses the potential of AI to revolutionize nursing education by providing personalized learning experiences while stressing the importance of ethical use and maintaining human interaction. In an umbrella review, Božić [16] suggested the need for comprehensive strategies to integrate AI into nursing education, including curriculum design and faculty development. Other researchers found that virtual simulations [17] and virtual reality (VR) technology [18] effectively develop nursing students’ knowledge and skills but do not develop critical reasoning. The researchers emphasized the need for more rigorous studies to confirm VR’s effectiveness in improving learning outcomes. They suggested that curriculum designers need to define the place and purpose of the pedagogy to ensure these simulations translate to real-world patient care. In a systematic review of studies on the effectiveness of virtual patient simulators for medical communication training, Lee et al. [19] found that evidence-based virtual patient simulator training enabled students to gain communication skills in a safe and affordable learning environment. Finally, Quay and Ramakrishnan [20] found that VR for immersive storytelling significantly improved empathy toward older adults. In summary, it is crucial to incorporate fundamental knowledge of AI technologies and their applications into nursing education. Moreover, efforts should be made to actively involve nurses in the technology research and development process [20]

This study was grounded in Kolb’s Experiential Learning Theory, which provides a framework for understanding the learning process [21]. The author conceptualized this process as a four-stage cycle: concrete experience (CE), reflective observation (RO), abstract conceptualization (AC), and active experimentation (AE). The CE stage involves engaging in an experience, while the RO stage focuses on deliberate reflection following that experience. The AC refers to deriving insights from the experience, and AE consists of applying those insights through action. A crucial element of Kolb’s theory is that learning is a continuous cycle, not a linear sequence, allowing learners to revisit and refine stages as they deepen their understanding and skills. There is little evidence to support using Kolb’s theory to support AI integration in nursing education. 

Given the many gaps presented above, this study aimed to evaluate the effectiveness of an Artificial Intelligence in Education (AIED) intervention in improving nursing students’ attitudes toward older adults. This aim is crucial as it addresses the global and national need to enhance nursing education with innovative AI-driven interventions, ensuring that future nurses are well-prepared to provide compassionate and competent care to the rapidly growing population of older adults.

## 2. Materials and Methods

### 2.1. Design

The researchers employed a convergent mixed-method pre/post-test study design to assess students’ attitudes toward older adults. Qualitative and quantitative data integration in this study followed a convergent mixed-method design, where both data types were collected and analyzed separately but concurrently. The purpose of this approach was to provide a comprehensive understanding of the impact of the AI-driven educational intervention on nursing students’ attitudes toward older adults. The study focused on senior-level community health nursing students in a southeastern United States baccalaureate program.

### 2.2. Sample Demographics

Enrollment in the community health nursing course was the inclusion criterion, while incomplete datasets were excluded from the study. Participation in the intervention was a course requirement for all students; however, they could opt out of the research component by providing signed consent. This approach ensured that while the educational activity was mandatory, students’ involvement in the study adhered to ethical standards, as approved by the university’s Institutional Review Board (IRB).

Since participation in the intervention was a course requirement for all students, the sampling technique was non-probabilistic, with the sample size being nearly 100% of the population. This minimized the impact of attrition and provided the study with the maximum feasible power, as additional recruitment was impossible. Therefore, traditional power analysis was considered unnecessary. The sample demographics included students’ self-identified gender and age.

### 2.3. Intervention

The community health nursing course implemented a three-phase approach to the AIED event, centered around Millie Larsen, an unfolding case from the National League for Nursing’s (NLN) Advanced Care for Seniors (ACES) series [22] (Table 1). The robust resources available through ACES provided evidence-based teaching materials that were free of charge and followed a patient throughout the care continuum. The Millie Larson case study was introduced in class and served as the basis for activities related to caring for older adults. The patient case served as the foundation for programming the AI patient simulator.

Phase One involved pre-class readings, a 90-min classroom session, and an independent review of the simulated patient’s medical record. A 22-item pre-simulation knowledge survey verified students’ understanding of the material.

Phase Two featured an AI-driven virtual simulation of Millie Larsen in a home environment. This non-immersive simulation allowed students to conduct a virtual patient interview utilizing advanced AI and natural language processing technology [23]. Faculty programmed the simulation to ensure the virtual patient provided accurate audio and visual responses aligned with the scenario learning objectives. If students pursued irrelevant questions, the simulator would respond with “I do not know what you mean” to encourage the students to redirect their questioning. Students interacted with the simulator on their personal computers using their microphones to engage in dialogue. Each student had an individual account, granted free of charge through an in-kind grant, allowing unlimited access to practice sessions at their convenience. Students typically spent approximately 30 min engaged with the AI-driven simulator. As part of this phase, students completed an abbreviated Outcome and Assessment Information Set Start of Care (OASIS) form. The OASIS form is a standardized data collection tool to assess the needs and outcomes of home health care patients. It includes various sections that evaluate the patient’s clinical, functional, and service needs, providing a comprehensive overview of their condition and care requirements [24]. After completing the OASIS, the participants drafted an email summarizing the patient encounter to a simulated multidisciplinary team.

Phase Three included an online Guided Reflection survey and an in-person debrief about the simulated experience. The in-person debrief lasted approximately 45 min and involved discussing the simulation experience, reflecting on their interactions with the virtual patient, and identifying key learning points. The Healthcare Simulation Standards of Best Practice guided the debriefing process and comprehensively evaluated the students’ experiences [25]. After the debrief, participants completed the National League for Nursing Simulation Design Scale [26] instrument. Students were given one week to access and complete the Phase Two and Three activities.

### 2.4. Instruments

#### 2.4.1. Data Collection

All instruments were administered via an online survey platform. Links to each instrument were available in the course learning management system. See Table 1.

#### 2.4.2. Attitudes

The UCLA Geriatric Attitude Scale (GAS) assessed nursing students’ attitudes toward aging before and after the educational intervention. This widely used tool evaluates perceptions of aging and older adults, offering valuable insights into respondents’ attitudes and beliefs. The GAS consists of five positively and nine negatively worded statements, rated on a 5-point scale from 1 (strongly disagree) to 5 (strongly agree), with higher scores reflecting more positive attitudes toward aging. The instrument demonstrated internal reliability with a Cronbach’s alpha of 0.76, which falls within the acceptable range of 0.7 to 0.9 [27]. Several studies have evaluated the GAS for reliability and validity. One study reported internal reliability with a Cronbach’s alpha of 0.78 and some evidence of content validity [28].

#### 2.4.3. Guided Reflection Survey

The authors composed a three-question, open-ended, Guided Reflection survey to gain insight into the student’s impressions of the learning event. The questions aimed to understand the impact on knowledge, skills, and attitudes after participating in the artificial intelligence in education intervention (AIED). Only the question related to attitudes was analyzed for this study.

### 2.5. Statistical Methods

#### 2.5.1. Quantitative Analysis

Quantitative data were collected using the GAS, administered to students before and after the intervention. This survey provided numerical data on students’ attitudes, which were analyzed using paired-sample *t*-tests to determine any statistically significant changes in attitudes pre- and post-intervention. Normality was checked using a quantile-quantile plot and the Shapiro–Wilk test. The *p*-value and effect size were reported to provide context for the results. All statistical analyses were performed using R version 4.3.2.

#### 2.5.2. Qualitative Analysis

For the qualitative data analysis, Atlas.ti [29] software was utilized to organize and interpret the text responses from question three of the Guided Reflection survey. The data were systematically coded using AI-driven software to identify key themes, and a hierarchical code system was developed to categorize recurring patterns. Advanced search and query functions were employed to explore relationships between codes and uncover co-occurring themes. Memos were written throughout the analysis process to document reflections and insights. Finally, reports were generated and exported to present the findings clearly and efficiently.

### 2.6. Ethical Considerations

The university’s Institutional Review Board (IRB) reviewed the study and determined it to be exempt. The study adhered to ethical principles, including independence, autonomy, confidentiality, and respect for participants. Participants were notified that completing the learning activity was mandatory for the course. However, informed consent was obtained from all participants, who were informed of their right to withdraw without affecting their course standing. Participants were treated with respect throughout the study and acknowledged for their contributions. All study-related data were de-identified and securely stored on password-protected computers. 

## 3. Results

### 3.1. Sociodemographic of Sample

There were 160 students enrolled in the course (*N* = 160). Only those who completed all the required data or did not provide consent were included in the study (*n* = 151), representing 94% of the total population. The demographic data are summarized in Table 2. The table shows that most participants identified as female (90%), with 74 students (49%) aged between 18 and 24 [30].

### 3.2. Instrument Reliability

The internal reliability of the GAS was Cronbach’s alpha = 0.76 for the pre-intervention scores and 0.78 for the post-intervention scores [30].

### 3.3. Mean Differences of Scores 

Since the study aimed to assess whether the AIED influenced nursing students’ attitudes toward older adults, a paired-sample *t*-test was conducted to compare pre- and post-experience scores. Only students with both pre- and post-experience scores were included in the analysis (*n* = 151). The results showed a slight increase in post-intervention scores (M = 35.07, SD = 5.34) compared to pre-intervention scores (M = 34.50, SD = 4.86). This modest improvement (d = 0.15) was statistically significant at the 0.10 level but not the 0.05 level (t = 1.88, *p* = 0.06). Also, considering the effect size in addition to the *p*-value allows for a more nuanced interpretation of the increase in score compared to sole reliance on the *p*-value [30].

### 3.4. Qualitative Results

The data from the Guided Reflection survey were analyzed using deductive content analysis. The emerging themes were challenging and overcoming ageism, increased empathy and patience, and enhanced communication skills (Table 3).

#### 3.4.1. Challenging and Overcoming Ageism

The undergraduate nursing students experienced a realistic encounter with an older adult, Millie Larson, in her home, raising students’ awareness of their attitudes toward older adults. Ageism was a key theme, which involved assuming someone is less capable or valuable due to age. Many students reported that the simulation allowed them to challenge and change their preconceived notions about older adults, helping them overcome ageist attitudes.

#### 3.4.2. Increased Empathy and Patience

After the AIED, students expressed more profound empathy and understanding of older adults’ unique needs and experiences. Consistent throughout the reflections was an awareness of more empathy. Students expressed self-realization of older adults’ daily life experiences. Also, students described their attitudes as changing towards being more patient. They acknowledged that the older adult had a longer story, and the learning activity allowed them to see first-hand the details of Millie Larson’s medical and social history.

#### 3.4.3. Enhanced Communication Skills

Students recognized that effective communication with older adults is a multifaceted skill that requires active listening, empathy, and a deep understanding of their unique needs. Many older adults want someone to listen to them, and student comments reflected this idea. Comments highlighted the importance of being fully present and attentive during interactions. Empathy also plays a significant role, as understanding older adults’ emotional and psychological needs can significantly enhance communication. Comments reflected the impact of empathy on creating a supportive environment where older adults feel valued and understood.

## 4. Discussion

The study aimed to evaluate the effectiveness of an Artificial Intelligence in Education (AIED) intervention in improving nursing students’ attitudes toward older adults.

The quantitative results showed a slight increase in post-intervention scores (M = 35.07, SD = 5.34) compared to pre-intervention scores (M = 34.50, SD = 4.86). This modest improvement (d = 0.15) was statistically significant at the 0.10 level (t = 1.88, *p* = 0.06). The data from the Guided Reflection survey were analyzed using deductive content analysis. 

The emerging themes were challenging and overcoming ageism, increased empathy and patience, and enhanced communication skills. 

The quantitative data showed a statistically significant increase in positive attitudes toward older adults; the qualitative data were examined to understand what contributed to this change, offering more profound insights into the reasons behind the shift in positive attitudes. This process involved looking for convergence (where qualitative and quantitative data supported each other), divergence (where they provided different perspectives), and complementarity (where they offered additional insights). The synthesis of findings involved combining the insights from both data sources to draw comprehensive conclusions.

The themes identified in the qualitative data were mapped against the quantitative results to compare the findings. There were no divergent or differing perspectives between the quantitative and qualitative findings. However, the data revealed both convergence and complementarity results. The quantitative data indicated a modest but statistically significant improvement in attitudes, further explained by the qualitative data showing that students developed greater empathy and patience through their interactions with the AI-driven simulation. This synthesis highlighted that while the numerical change in attitudes was modest, the students’ qualitative experiences provided a richer understanding of the intervention’s impact.

The findings from this mixed-method study underscore the significant impact of AI-driven educational interventions on nursing students’ attitudes toward older adults. The quantitative results from the GAS reveal a statistically significant improvement in students’ attitudes post-intervention. This indicates that the AIED event effectively enhanced their perceptions and readiness to care for older adults. As highlighted in the study’s background, this aligns with the urgent need to prepare nursing students for the projected increase in the older adult population [1,2].

The background of this study emphasizes the critical gap in geriatric education within nursing programs, which has profound implications for the quality of care older adults receive. The American Academy of Colleges of Nursing [31] guidelines stress the importance of integrating geriatric competencies into nursing curricula. However, many programs need to improve in this area, leading to a need for more confidence and competence among nursing students. This study’s positive shift in attitudes suggests that AI-driven simulations can be a powerful tool in bridging this educational gap. By providing realistic and engaging learning experiences, the AIED event helps students develop the necessary skills and confidence to care for older adults effectively.

Ageism and negative attitudes toward older adults are significant barriers to quality care. The qualitative findings from this study reveal that the AI-driven simulation helped students challenge and overcome their ageist attitudes. Students reported a newfound respect and empathy for older adults, recognizing their unique needs and capabilities. This change is crucial, as research indicates that ageism in healthcare settings can lead to poorer health outcomes for older adults [7]. These findings support earlier studies addressing ageism in nursing education [3,11,12,13,14]. Addressing these biases early in nursing education can foster a more respectful and empathetic approach to older adult care, which may improve patient outcomes. The qualitative findings from this study reveal that the AI-driven simulation helped students challenge and overcome their ageist attitudes, fostering a newfound respect and empathy for older adults.

Effective communication is a cornerstone of quality care for older adults. The qualitative data highlight that students experienced significant improvements in their communication skills, particularly in active listening, empathy, and patience. These skills are essential for building therapeutic relationships and providing personalized care. The AI-driven simulation allowed students to practice and refine these skills in a safe and supportive environment. This aligns with previous research, such as the systematic review by Lee et al. [10], who found that virtual patient simulators can effectively enhance communication skills in medical training. 

Integrating AI-driven simulations into nursing education offers a promising avenue for enhancing students’ competencies in geriatric care. The findings from this study support the recommendations of Harder [32] and Von Gerich et al. [33] to advance healthcare simulation through AI-driven tools. These simulations provide personalized feedback and can simulate diverse patient interactions, helping students develop a deeper understanding of the complexities of geriatric care. By incorporating these innovative technologies into nursing curricula, we can better prepare students to meet the challenges of an aging population. 

## 5. Limitations and Future Research

This study has several limitations. First, AI technology integration in clinical simulation includes new technologies. Therefore, little is known about its strengths and limitations. New theoretical models for integrating the technologies into clinical simulation are needed to support sound educational practices. Additionally, the rapid advancement of AI technologies has outpaced the development of comprehensive policies and regulations. For example, ethical guidelines for accountability, privacy, and data protection are needed. These policies should be addressed and integrated into best practice standards. Second, the study used a single AIED session at one point in the nursing curriculum. It would be valuable to investigate the impact of AIED at various stages of the curriculum, as repeated exposure to simulated learning events may provide insight into whether changes in attitudes toward older adults are retained. Third, the impact of the AIED event was isolated to the nursing discipline. Exploring the efficacy of a three-phased AIED event across healthcare disciplines such as Physical Therapy, Nutritional Services, Pharmacy, and Medicine would be beneficial. Lastly, future research should explore how these findings translate to participants’ understanding of older adults in clinical encounters.

## 6. Practical Implications

The results of this study suggest that incorporating AI-driven simulations into nursing education can significantly influence policy decisions by demonstrating the effectiveness of innovative teaching methods. These findings advocate for integrating AI technologies in curriculum development, ensuring that nursing education practices align with healthcare’s evolving needs. By adopting AI-driven simulations, educational institutions can enhance the learning experience, better prepare students for real-world challenges, and ultimately improve the quality of care provided to older adults.

## 7. Conclusions

Integrating qualitative and quantitative data, the study provided a nuanced understanding of how the AI-driven educational intervention influenced nursing students’ attitudes toward older adults. The combined analysis highlighted the intervention’s strengths and areas for further development, ultimately contributing to the goal of preparing nursing students to provide compassionate and effective care to an aging population.

As the population of older adults continues to grow, it is imperative to address the educational gaps in geriatric care and equip future nurses with the skills and attitudes necessary to provide compassionate and effective care. AI-driven simulations represent a valuable tool in achieving this goal, offering realistic and engaging learning experiences that can significantly enhance students’ readiness to care for older adults. Future research should continue to explore the impact of AI-driven simulations on nursing education and identify best practices that will significantly impact a rapidly aging population.

## Figures and Tables

**Table 1:** Study summary.

Intervention		
Pre-Intervention Assessment	Theoretical Constructs	Data Collection Method
Geriatric Attitude Survey	AC	Online survey
Phase One: Pre-AIED Event Activities		
1. Pre-class readings	AC, RO	N/A
2. Ninety-minute interactive classroom session	AC, RO, AE	N/A
3. Pre-simulation electronic medical record review	AC, RO, AE	N/A
4. Pre-simulation knowledge survey	AC, RO	Online survey
Phase Two: AIED Event Activities		
1. AI-driven virtual non-immersive simulator experience	CE	N/A
2. Completion of OASIS form	AC, RO, AE, CE	Online survey
3. Create report email to multidisciplinary team	AC, RO, AE, CE	Online document submission
Phase Three: Post-AIED Activities		
1. Guided Reflection survey/debrief	RO	Online survey
2. NLN Simulation Design Scale completion	RO	Online survey
Post-Intervention Assessment		
UCLA Geriatric Attitude Survey	AC	Online survey

Theoretical constructs key: Concrete Experience (CE), Reflective Observation (RO), Abstract Conceptualization (AC), Active Experimentation (AE).

**Table 2:** Demographics.

Category	Frequency	Percentage
*n* = 151		
Gender		
Male	15	10%
Female	136	90%
Age Range		
18–24	74	49%
25–34	59	39%
35–44	11	7%
45–54	6	4%
55–64	1	1%
65+	0	0%
Mean	27.7	
Median	25	
Standard Deviation	7.5	

**Table 3:** Qualitative data to support themes.

Qualitative Theme	Participant Comment
Challenging and Overcoming Ageism	“I had some bias going into the simulation… However, Millie was the opposite. She was very kind and even funny. She was patient and responsive”. “Ageism is a bias we cannot participate in. Older adults can teach us so much about life! They have so much knowledge about the world. I didn’t realize how much I would enjoy talking to older adults after this simulation”. “I used to not want to take care of older adults since I believed they would be more difficult patients to work with, but this simulation has changed my mind. I now know that older adults just need some extra patience and time in order to deliver the best care to them, as their bodies and minds are in a different stage than mine”. “My attitude has changed and fully rejects the negative stereotypes about aging. I don’t see older patients the same way anymore; now, I see them as people who deserve the best care possible because they already contributed so much to society. Older adults have so much wisdom to share, and now I am very excited to listen to their stories”. “While I already had a fairly positive attitude towards older adults, participation in this simulation has further improved my view of caring for older adults as patients. …I look forward to caring for older adults…”
Increased Empathy and Patience	“I never realized how much older adults go through daily. This experience opened my eyes to their struggles and strengths”. “I am more empathetic and understanding about the changes and hardships older adults go through”. “By participating in this simulation, my attitude towards working with older adults is more patient, more understanding, and more caring with this population”.
Enhanced Communication Skills	“I have learned that they just want someone to listen to them”. “Building a rapport and getting to know your older adults is especially important because they sometimes aren’t forthcoming with all of their feelings or symptoms”. “I see that older adults may have some cognitive decline, but with patience, the goals can still be achieved”. “The AI training emphasized the importance of clear and respectful communication. I feel more confident in my ability to connect with older patients”. “Understanding that sometimes the older people tend to give more information than is necessary, but to actively listen anyway”. “I learned that listening is just as important as speaking. Older adults have stories and experiences that are crucial to their care”.

## Data Availability

The data presented in this study are available on request from the corresponding author.

## Public Involvement Statement

There was no public involvement in any aspect of this research.

## Guidelines and Standards Statement

This manuscript was drafted against the SQUIRE-ED (Standards for Quality Improvement Reporting Excellence in Education: Publication Guidelines for Educational Improvement) for quantitative research.
